# Pre-eclampsia is associated with altered expression of the renal sodium transporters NKCC2, NCC and ENaC in urinary extracellular vesicles

**DOI:** 10.1371/journal.pone.0204514

**Published:** 2018-09-24

**Authors:** Chih-Chiang Hu, Marina Katerelos, Suet-Wan Choy, Amy Crossthwaite, Susan P. Walker, Gabrielle Pell, Mardiana Lee, Natasha Cook, Peter F. Mount, Kathy Paizis, David A. Power

**Affiliations:** 1 Melbourne Medical School, University of Melbourne, Parkville, Victoria, Australia; 2 Kidney Laboratory, Institute for Breathing and Sleep (IBAS), Austin Health, Heidelberg, Victoria Australia; 3 Department of Nephrology, Austin Health, Heidelberg, Victoria, Australia; 4 Mercy Hospital for Women, Heidelberg, Victoria, Australia; 5 Department of Medicine, University of Melbourne, Parkville, Victoria, Australia; 6 Obstetrics and Gynecology, University of Melbourne, Parkville, Victoria, Australia; Universidad de la Laguna, SPAIN

## Abstract

Pre-eclampsia is a hypertensive disorder of pregnancy characterised by hypertension and sodium retention by the kidneys. To identify changes in sodium uptake proteins in the tubules of the distal nephron, we studied their expression in urinary extracellular vesicles or exosomes (uEVs). Urine was collected from women with pre-eclampsia or during normal pregnancy, and from healthy non-pregnant controls. uEVs were isolated by centrifugation and analyzed by Western blot. Expression, proteolytic cleavage and phosphorylation was determined by densitometric analysis normalized to the exosome marker CD9. Results showed a significant increase in phosphorylation of the activating S130 site in NKCC2, the drug target for frusemide, in women with pre-eclampsia compared with normal pregnant women. Phosphorylation of the activating sites T101/105 in NKCC2 was similar but the activating T60 site in NCC, the drug target for thiazide diuretics, showed significantly less phosphorylation in pre-eclampsia compared with normal pregnancy. Expression of the larger forms of the α subunit of ENaC, the drug target for amiloride, was significantly greater in pre-eclampsia, with more fragmentation of theγ subunit. The differences observed are predicted to increase the activity of NKCC2 and ENaC while reducing that of NCC. This will increase sodium reabsorption, and so contribute to hypertension in pre-eclampsia.

## Introduction

Pre-eclampsia complicates 3–8% of pregnancies resulting in significant maternal, fetal and neonatal morbidity and mortality [1]. The multisystem manifestations of pre-eclampsia occur after 20 weeks gestation with common clinical features including hypertension and proteinuria [2]. The pathogenesis of pre-eclampsia involves placental release of soluble fms-like tyrosine kinase (sFlt-1), a non-membrane-associated circulating form of the receptor for vascular endothelial growth factor (VEGF), which inhibits endothelial VEGF signalling leading to reduced nitric oxide synthesis, endothelial injury, endotheliosis, glomerular dysfunction and proteinuria [3]. Generalized edema is a common manifestation of pre-eclampsia, with proteinuric patients displaying avid sodium retention, which occurs despite suppression of the renin-angiotensin-aldosterone system and intravascular contraction [4, 5].

Although the sodium transporters responsible for sodium retention in pre-eclampsia are unknown, the most important transporters affecting renal sodium reabsorption in inherited disorders of hypo- or hypertension are the Na-Cl2-K co-transporter 2 (NKCC2), the Na-Cl co-transporter (NCC) and the epithelial sodium channel (ENaC) [6]. These proteins are found on the apical surface of unique areas of the distal nephron, and are the drug targets for loop diuretics, thiazide diuretics, and amiloride, respectively. NKCC2 and NCC are activated by phosphorylation, which is associated with surface expression and regulated primarily by the WNK-SPAK/OSR-1 pathway [7]. SPAK and OSR-1 phosphorylate NKCC2 on the T101 and 105 sites in the intracellular N-terminus of the molecule [8]. Phosphorylation of T105 increases co-transporter activity in vitro whereas phosphorylation of T101 has little effect [9]. NKCC2 is also phosphorylated on S130 by protein kinase A (PKA) and, to a lesser extent, the energy sensing kinase AMPK [10, 11]. S130 is the second major activating phosphorylation site in the N-terminus of NKCC2 [9]. Mutation of both T105 and S130 renders the co-transporter inactive[9]. NCC is phosphorylated at three residues by SPAK and OSR-1, but the T60 phosphosite appears to be the most important for co-transporter activity [12].

By contrast, ENaC activity is determined by cell surface expression and regulation of channel open probability, which is influenced by activating proteolytic cleavage of the α and γ subunits [13–15]. The α subunit is activated by intracellular furin-mediated cleavage at two sites in the N-terminus, which removes an inhibitory domain [16]. There are also less well-characterised potential cleavage sites for extracellular proteases. The γ subunit is cleaved once by intracellular furin [16]. Further extracellular cleavage by proteases occurs C-terminal to the initial site and removes a 43-amino acid domain, leaving an approximately 50 kD subunit detected by C-terminal antibodies. The γ subunit is subject to a number of other, less well-characterised proteolytic events by a range of proteases, potentially generating smaller molecular weight forms.

We have previously shown that development of obesity-related hypertension in mice is associated sequentially with increased expression of NCC, followed by increased phosphorylation of S130 and T101/105 (using human amino acid numbering) [17, 18]. Changes in sodium transporters in the distal nephron have not been well-studied in human pregnancy. Nielsen et al reported that the abundance of the α subunit of ENaC was increased in normal pregnancy, particularly a 50 kD species, but they were unable to identify any differences in expression of either the α or γ subunits between women with normal pregnancies and those with pre-eclampsia [19].

Previously, the expression and activity of sodium transporters has been difficult to study *in vivo* in human subjects. It has been reported, however, that apical transporters appear in the urine through the secretion of uEVs, which are membrane vesicles that originate as internal vesicles of multivesicular bodies [20–23]. We hypothesized that hypertension in pre-eclampsia is associated with altered expression and activity of renal tubular sodium transporters and that these changes will be detectable and measurable in urinary uEVs. To study this question, we examined expression and phosphorylation of sodium transport proteins from the distal nephron in urinary uEVs from subjects with pre-eclampsia, and compared it with uEVs from women during normal pregnancy and healthy women of reproductive age.

## Methods

### Study design and participant groups

A cross-sectional, non-interventional study of adult subjects >18 years old was conducted in two tertiary referral centres (Austin Health and Mercy Hospital for Women). The study was approved by the Human Research Ethics Committees of Mercy Health and Austin Health. All subjects gave written informed consent prior to participation in the study. Patients with pre-eclampsia and normotensive pregnant controls were recruited from clinics and inpatient units at Mercy Hospital for Women. Healthy non-pregnant female subjects were recruited from medical student volunteers at the Austin Health Clinical School, University of Melbourne. The period of recruitment was from February 2015 to December 2016. At the time of recruitment, demographic information was obtained from participants, including age, gestation and parity in pregnant groups, body mass index, medical history and current medications.

Patients were included in the pre-eclampsia group if they met the following criteria: new onset of hypertension after 20 weeks of gestation, which was defined as office seated systolic blood pressure ≥140mmHg and/or diastolic blood pressure ≥90mmHg, plus an additional clinical or biochemical feature of renal, haematological, hepatic, neurological or fetal involvement as per the guidelines of the Society of Obstetric Medicine of Australia and New Zealand (SOMANZ) and the International Society for the Study of Hypertension in Pregnancy (ISSHP) [2, 24].

Criteria for the normotensive pregnant group were: (a) seated office blood pressure <140/90mmHg, (b) no systemic features of pre-eclampsia, and (c) in the third trimester of their pregnancy. Healthy female volunteers were predominantly medical students recruited from Austin Health Clinical School (University of Melbourne). Exclusion criteria for the healthy control group were pregnancy, any history of hypertension, BP ≥ 140/90, and any history of kidney disease.

### Measurement of blood pressure

Study participants were rested for at least 5 minutes in a seated position with the arm supported at heart level prior to having measurements taken with an aneroid sphygmomanometer (Welch Allyn, NY, USA). Two readings of blood pressure were obtained from each arm and the overall average was recorded in the data entry form.

### Urine collection, processing and storage

Morning urine samples (up to 40 ml) were collected from study subjects in sterile containers. 1 ml of urine was removed and frozen at -80° C for biochemical analysis. Sigma Protease Inhibitor (PI) Cocktail (Sigma-Aldrich, Missouri, USA; 125μl PI/ 10ml urine) was added immediately to the remaining sample. Samples were then centrifuged at 1,600xg for 10 min at 4°C to remove whole cells, large membrane fragments and other debris. Supernatants were stored at -80° C until processed.

### Isolation of urinary uEVs by differential centrifugation

UEVs were isolated from urine by centrifugation as described [25]. Urine samples (9ml) were thawed by continuous vortexing, aliquoted into three 3ml polycarbonate tubes and centrifuged (Beckman Optima benchtop ultracentrifuge) at 17,000xg for 10 min at 4°C. Supernatants were centrifuged at 200,000xg for 1h at 4°C and crude exosome pellets were recovered. The pellet of each tube was re-suspended in phosphate buffered saline (PBS) supplemented with PI (125μl PBS/ 10μl PI), pooled into one clean tube and more PBS/PI solution was added to a final volume of 3ml. The sample was centrifuged again at 200,000xg for 1h at 4°C. The exosome pellet was re-suspended in 50μl of PBS/PI solution. The sample (41μl) was transferred to an Eppendorf tube with the addition of 12μl of Laemmli sample buffer containing SDS, glycerol and dithiothreitol to final concentrations of 1.5%, 6% and 60mg/ml, respectively, in a final volume of 60μl. Samples were stored at -80°C until use.

### Western blotting

Exosome samples were heated at 95°C for 5 min and resolved on 4–15% precast gradient gels (Bio-Rad, California, USA). The proteins were then transferred electrophoretically to polyvinylidene fluoride (PVDF) membranes, and processed as we have previously described [11]. Blots were cut at approximately 100 kD so that the two halves could be blotted separately. Immunoreactive proteins were detected by enhanced chemiluminescence with the SuperSignal Chemiluminescent System (Perkin Elmer). Western blots were quantified by densitometry (Scion Image for Windows, Scion Corporation, Frederick, Maryland). If the membrane was to be probed with another primary antibody, antibody bound to the membrane was stripped by an incubation in Reblot stripping solution (Chemicon) for 15 min. Quantification of Western blots was performed by densitometry with analysis using ImageJ software [National Institutes of Health (NIH), Bethesda, MD].

Densitometric data is shown in the Figures as box-and-whisker plots (middle line in the box is the median value, with box limits at the 25^th^ and 75^th^ percentile; whiskers extend to include all values). Variability of the assays was estimated using a sample from a normal control analysed on 3 separate occasions (S1 Fig). This was not found to be excessive.

### Primary antibodies

Rabbit polyclonal antibodies against phospho-NKCC2-S130 (pS130), phospho-NKCC2-T101/105 (pT101/105) and phospho-NCC-T60 (pT60) were produced and validated by our laboratory as previously described [26, 27]. Rabbit polyclonal antibodies were purchased commercially for total NKCC2, ENaC-alpha and ENaC-gamma (Stress Marq, British Columbia, Canada), NCC (Abcam, Cambridge, UK) as well as a rabbit mAb against CD9 (Cell Signaling Technology, Massachusetts, USA) (Table 1).

**Table 1:** Antibodies used in the study.

Antibody	Species	Immunising antigen	Source
Anti-NKCC2	Rabbit polyclonal	Rat N-terminus aa 33–55	Stress-Marq
Anti-pNKCC2-T101/105	Rabbit polyclonal	Human N-terminus aa 99–110	Fraser et al [26]
Anti-pNKCC2-S130	Rabbit polyclonal	Mouse N-terminus aa 121–130	Cook et al [27]
Anti-NCC	Rabbit polyclonal	Rat N-terminus aa 74–95	Abcam
Anti-pNCC-T60	Rabbit polyclonal	Human N-terminus aa 54–66	Fraser et al [26]
Anti-α-ENaC	Rabbit polyclonal	Rat N-terminus aa 46–68	Stress Marq
Anti-γ-ENaC	Rabbit polyclonal	Rat C-terminus aa 629–650	Stress Marq
Anti-CD9	Rabbit monoclonal	Human around aa 178	Cell Signaling Technology

### Urinary electrolytes

These were performed using an autoanalyzer. The concentration of urinary sodium was not determined below 20 mmol/L and chloride below 60 mmol/L.

### Statistical analysis

Using Image J software, band densities were quantified and given individual densitometry values. If no band was detectable then the densitometry was recorded as zero. Correction for different exposures across blots was made by dividing the value of each sample with the average of all samples probed by the specific primary antibody. Data were tested for normal distribution and, in all instances, the data were not normally distributed and the Kruskall-Wallis test was used to establish a difference between groups. Dunns’ multiple comparison test was then used to identify specific differences in the data. Expression of transporters was normalised to the expression of the tetraspanin CD9, which was used as an exosome marker [28]. Phosphorylation of transporters was determined as a ratio of expression of the protein and CD9. These statistical analyses were performed using GraphPad InStat v3 software. A ‘p’ value < 0.05 was considered significant.

In a second analysis, bands on Western blots were scored as present or absent and the number of individuals with visible bands in each of the groups compared with Chi-square or 3-way Fisher’s exact test. This calculation was performed using the webpage Vassarstats.net. Provided the result was significant (p<0.05) comparisons were then made using a 2-way Fisher’s exact test between each of the groups and a Bonferroni correction for the number of comparisons made (n = 3).

Linear regression was performed in the PE group, using Microsoft Excel and GraphPad, to determine whether there was any correlation between the parameters noted. The regressions made involved all of the Western blot data as densitometric ratios with CD9, systolic and diastolic blood pressures, and urinary protein/creatinine ratio.

## Results

### Subject characteristics

A total of 24 women with pre-eclampsia (the PE group), 24 normotensive pregnant women (the NP group) and 21 non-pregnant normotensive women (the NC group) were recruited for the study. The characteristics of all of the study subjects are summarised in Table 2. One subject in the NC group had missing pNKCC2 and pNCC data.

**Table 2:** Characteristics of subjects in the PE, NP and NC groups at enrolment.

Study groups	Pre-eclampsia N = 24	Normotensive pregnant N = 24	Non-pregnant control N = 21	P-value (PEvsNP)
**Age (yrs)**	31.0±5.4	31.8±3.9	25.1±1.3	n.s.
**Gestation (week)**	31.6±3.4	32.2±3.7	n/a	n.s.
**Primiparous (%)**	58	38	n/a	n.s.
**SBP**^**1**^ **(mmHg)**	151.5±13.8	113.1±7.8	104.3±10.5	<0.001
**DBP**^**2**^ **(mmHg)**	92.7±8.2	68.1±7.9	69.0±8.0	<0.001
**BMI**^**3**^ **(kg/m**^**2**^**)**	28.8±8.6	29.5±6.6	20.5±1.9	n.s.
**Urine Pr/Cr (mg/mmol)**^**4**^	220 (11–896)	10 (6–14)	6 (4–13)	<0.001

^1^Systolic blood pressure

^2^Diastolic blood pressure

^3^Body mass index

^4^Urine Pr/Cr was done on 23 subjects in the PE group, 17 in the NP group, and 21 in the NC group.

Data are expressed as mean ± standard deviation if parametric or median (IQR) if not parametric. n.s. = not significant

### NKCC2 expression and phosphorylation

**Fig 1:**
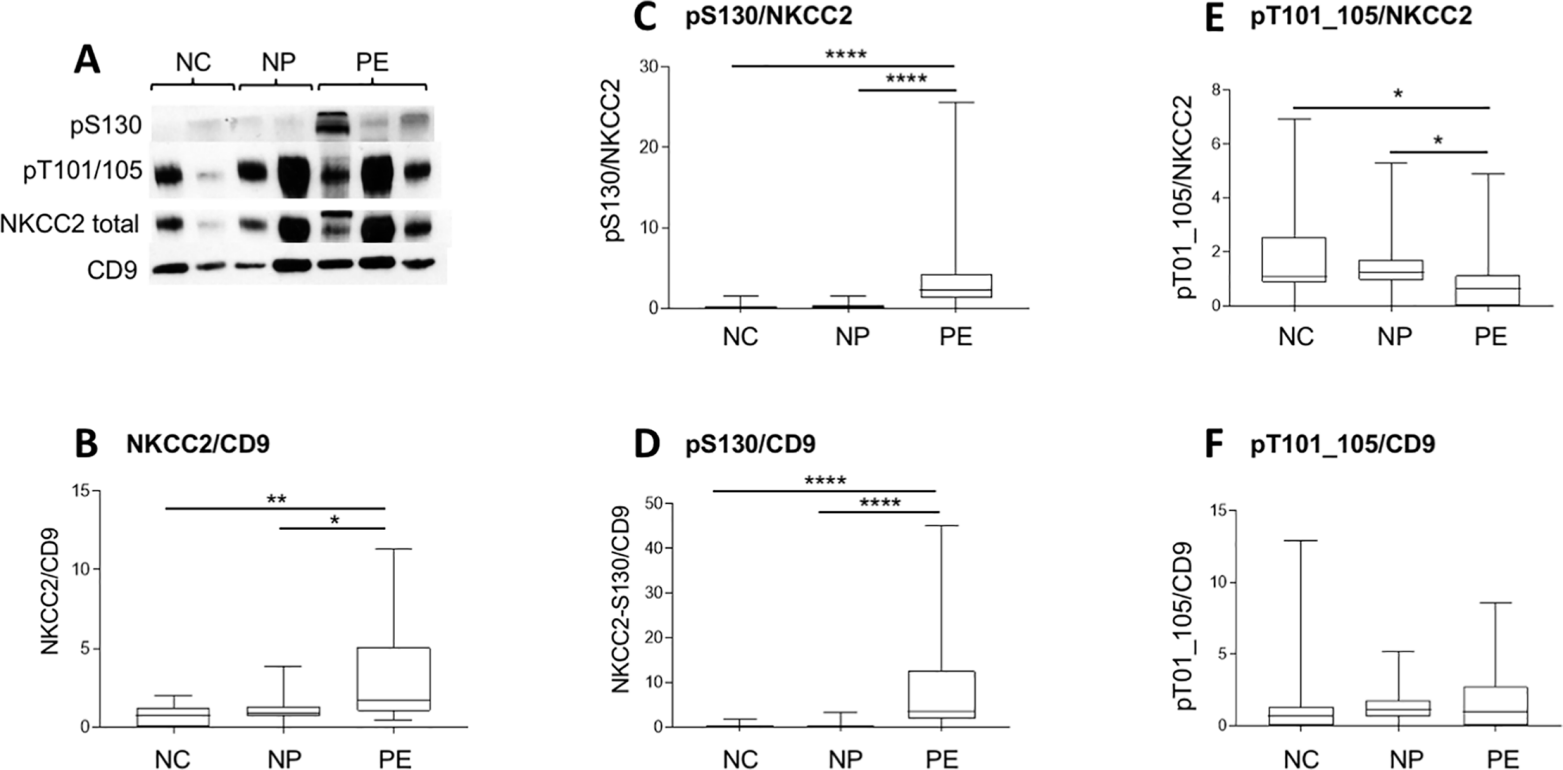
Western blot and densitometry showing expression of NKCC2. (A) Representative Western blot from normal controls (NC), women with normal pregnancies (NP) and with pre-eclampsia (PE) probed with antibodies against NKCC2, pNKCC2 (S130 and T101/105), and CD9. (B) Densitometric analysis of total NKCC2 normalised for CD9 in the three groups. Difference between groups by Kruskall-Wallis test p<0.002. (C and D) Densitometric analysis of total NKCC2 pS130 normalised for total NKCC2 (C) and CD9 (D) in the three groups. Difference between groups by Kruskall-Wallis test p<0.0001 for both comparisons. (E and F) Densitometric analysis of total NKCC2 pS130 normalised for total NKCC2 (E) and CD9 (F) in the three groups. Difference between groups by Kruskall-Wallis test p<0.01 for (E) and not significant for (F). Individual comparisons by Dunns’ test *p<0.05, **p<0.01, ****p<0.0001.

NKCC2 was detected at approximately 150 kD on Western blots (Fig 1A). A probable dimeric form at >200 kD was not quantitated. The level of NKCC2 expression was significantly different between groups with greater expression in the PE group compared with NP (p<0.05; Fig 1B). There was significant more phosphorylation of NKCC2 on S130 in the PE group, whether expressed as a ratio of total NKCC2 or as a ratio of CD9 (p<0.001; Fig 1C and 1D). Visible bands for pS130 were seen in 23/24 subjects with PE, compared with 6/24 and 6/20 subjects in the NP and NC groups, respectively (p<0.001; Table 3). The difference between PE and NP was significant (p<0.001, 2x2 Chi-square with Bonferroni correction). Phosphorylation of the SPAK/OSR1 phosphosites T101/105 was less in the PE group when expressed as a ratio of total NKCC2 (p<0.05) but not CD9 (Fig 1E and 1F). There was no difference in the number of subjects in each group showing a visible band for T101/105 (Table 3).

**Table 3:** Detection of bands for phosphorylated forms of NKCC2 and NCC, and for ENaC proteins.

Species	PE	NP	NC	P-value*
**NKCC2-pS130**	23/24	6/24	6/20	<0.001^#^
**NKCC2-pT101/105**	15/24	21/24	16/20	n.s.
**NCC -pT60**	11/24	21/24	18/20	<0.001^#^
**α-ENaC (total)**	20/24	12/24	13/21	<0.05^#^
**α-ENaC 90–100 kD**	2/24	3/24	4/21	n.s.
**α-ENaC 75 kD**	18/24	2/24	2/21	<0.001^#^
**α-ENaC 50 kD**	14/24	7/24	11/21	n.s.
**γ-ENaC (total)**	18/24	15/24	12/21	n.s.
**γ-ENaC 90–100 kD**	0/24	5/24	3/21	n.s.
**γ-ENaC 75 kD**	9/24	8/24	8/21	n.s.
**γ-ENaC 57–62 kD**	11/24	1/24	0/21	<0.001^$^
**γ-ENaC 50 kD**	8/24	1/24	4/21	n.s.

The numerator is the number with a detectable band, and the denominator is the total number of samples analysed. One NC subject with missing pNKCC2 and pNCC data. n.s. = not significant.

*P-value refers to a 2x3 way contingency table comparing all groups analysed by or

^#^Chi-square or

^$^Fisher’s exact test.

### NCC expression and phosphorylation

**Fig 2:**
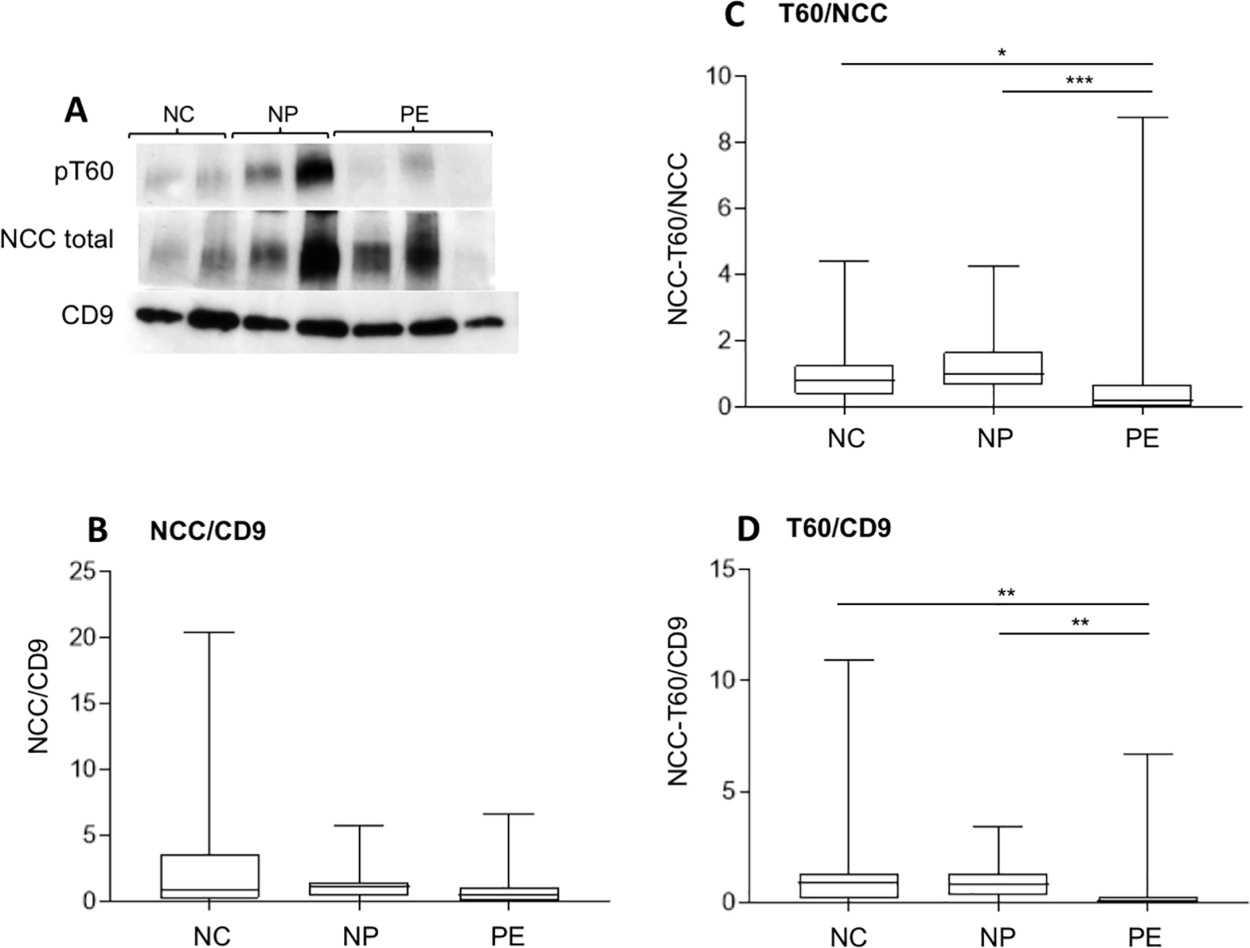
Western blot and densitometry showing phosphorylation of NCC and pNCC. (A) Representative Western blot from normal controls (NC), women with normal pregnancies (NP) and with pre-eclampsia (PE) probed with antibodies again NCC-pT60, NCC, and CD9. (B) Densitometric analysis of total NCC normalised for CD9 in the three groups. No difference between groups by Kruskall-Wallis test. (C and D) Densitometric analysis of total NCC pT60 normalised for total NCC (C) and CD9 (D) in the three groups. Difference between groups by Kruskall-Wallis test p<0.001 for (C) and p<0.002 for (D). Individual comparisons by Dunns’ test *p<0.05, **p<0.01, and ***p<0.001.

NCC ran at approximately 150 kD and, again, probable dimeric forms seen at a higher MW were not quantitated. There was no difference between the groups in expression of NCC (Fig 2A and 2B), but phosphorylation of the SPAK/OSR1 site at T60 was significantly less in the PE group compared with normal pregnancy (p<0.001) and normal controls (p<0.05; Fig 2C). When expressed as a ratio of CD9, the reduction in T60 phosphorylation in women with pre-eclampsia was still present (p<0.01; Fig 2D). Detectable phosphorylation of T60 was seen in 11/24 in the PE group, 21/24 in the NP group and 18/20 in the NC group (p<0.001; Table 2).

### ENaC expression in urinary uEVs

#### α-ENaC subunit

**Fig 3:**
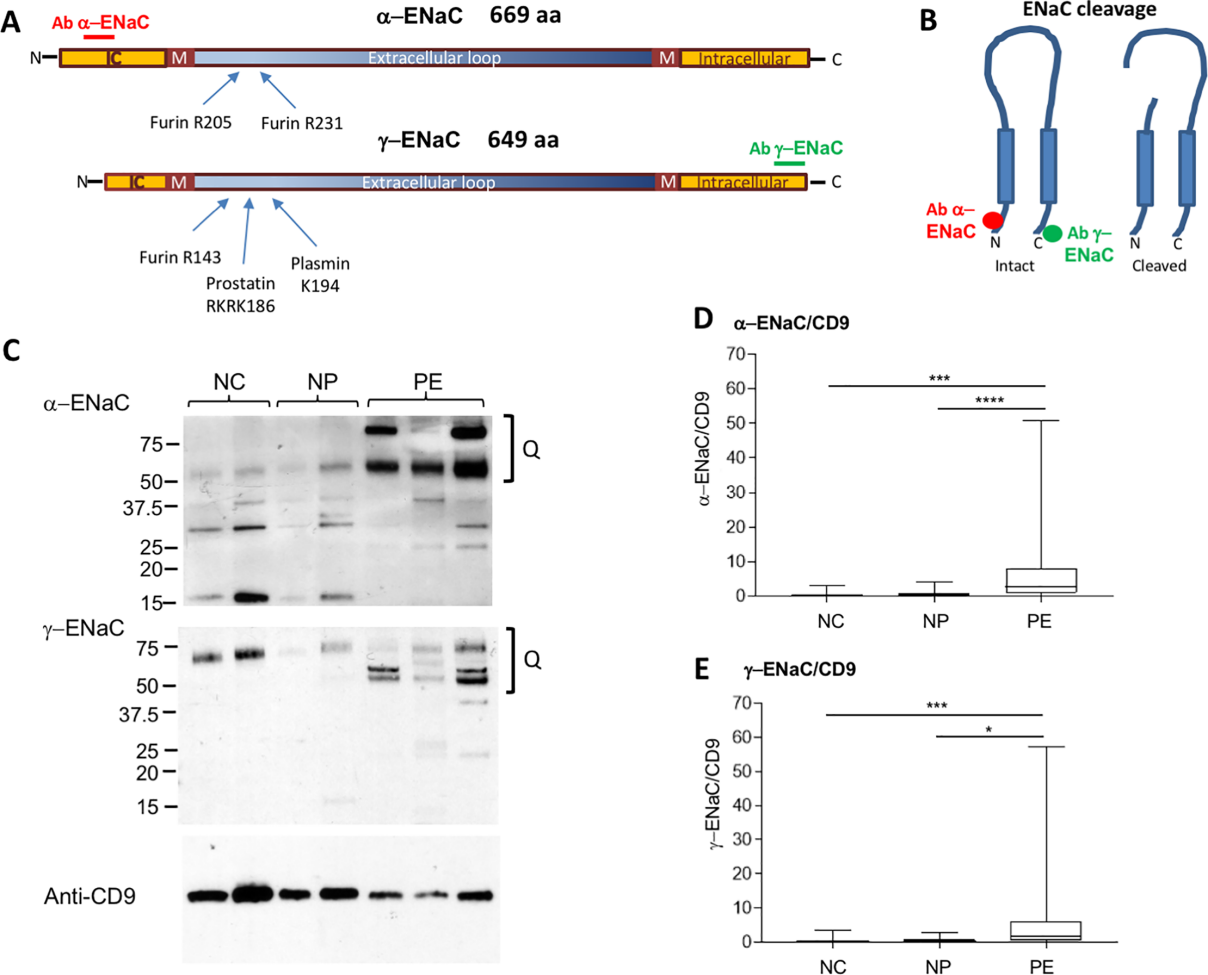
Expression of α-ENaC and γ-ENaC by Western blot. (A) Schematic of human α-ENaC and γ-ENaC, modified from Kleyman et al [16]. Antibody binding sites, based on similarity to the rat sequence used to generate the original antibody is shown. For human α-ENaC it was 78% identical to the rat sequence over amino acids 20–42, while for γ-ENaC it was 86% identical over the residues 628–649. M = predicted transmembrane region (derived using DAS-TMfilter). Intracellular (IC) and extracellular regions shown. (B) Effect of cleavage on the α- or β-subunit of ENaC. Predicted antibody binding sites shown. (C) Representative Western blot from normal controls (NC), women with normal pregnancies (NP) and with pre-eclampsia (PE) probed with antibodies again α-ENaC, γ-ENaC, and CD9. The symbol Q in (C) represents the area of the blot used in densitometry. (D and E) Densitometric analysis of total α-ENaC (D) and γ-ENaC (E) normalised for CD9 in the three groups. Difference between groups by Kruskall-Wallis test p<0.0001 for (D) and p<0.005 for (E). Individual comparisons by Dunns’ test *p<0.05, ***p<0.001 and ****p<0.0001.

The organisation of α-ENaC and proteolytic sites are shown in Fig 3A and 3B. The antibody used in this study was directed against amino acids 46–68 at the rat protein N-terminus (Fig 3B). Multiple bands from 50–100 kD were quantitated together and significantly greater in PE compared to both NP and NC (p<0.001; Fig 3C and 3D). A major band at 75 kD was seen in 18/24 in the PE group, 2/24 in the NP group and 2/21 in the NC subjects (Table 3). The difference between PE and NP was significant (p<0.001). There were also bands at 50 and 90–100 kD seen in some subjects from all groups (Table 3), but their presence did not differ between groups. There were a number of lower MW bands seen in all groups. These occurred at 15, 25, 30, 32, 35, 37.5 and 40 kD. They were present in 13/24 PE, 9/24 NP and 7/21 NC subjects. There was no discernible pattern to their appearance or obvious difference between groups.

#### γ-ENaC subunit

The organisation of α-ENaC and proteolytic sites are shown in Fig 3A and 3B. Using an Ab directed against the C-terminus of γ-ENaC, major bands of 75, 57–62 and 50 kD were seen (Fig 3C). Combined evaluation of all three bands by densitometry showed a significant difference between the PE and NP groups (p<0.05), as well as a more marked difference between PE and NC (p<0.001; Fig 3E). The 75 kD band was present in similar numbers of subject in each of the three groups (Table 3). Bands between 57–62 kD, however, were significantly different between groups (p<0.001; Table 3). There was a significantly greater number of women in the PE group showing these bands compared with the NP group (p<0.01). The 50 kD band was seen in 8/24 subjects in the PE group but was not different between groups (Table 2).

#### Urinary electrolytes

Urinary electrolytes were available for 23, 17 and 24 subjects in the PE, NP and NC groups, respectively, including a few subjects who did not have Western blot data. There were no differences in urinary Na/Cr or K/Cr ratios between the three groups. There were 4/23 subjects in the PE group and 4/21 in the NC group with urinary sodium levels below the limit of detection. Undetectable values for urinary chloride occurred in 15/23 subjects in the PE group, 6/17 in the NP group and 9/24 of the normal controls. There was a trend to a difference between the groups for urine chloride but it was not significant (p = 0.09; Chi-square).

### Linear regression analysis in women with PE

**Fig 4:**
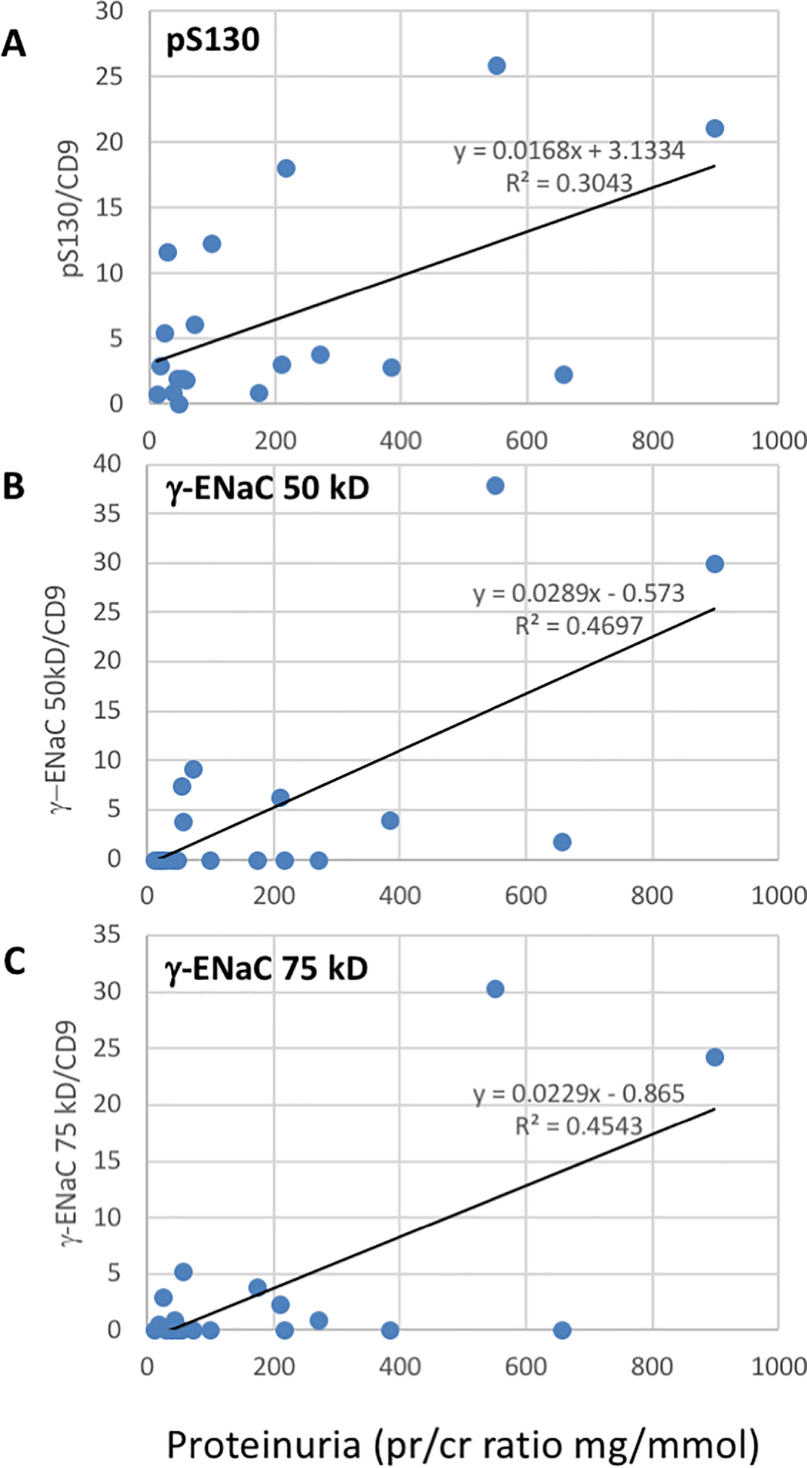
Linear regression analysis of proteinuria and sodium transporter band intensities in women with PE. (A) NKCC2 pS130 vs urine protein/creatinine ratio, p = 0.014, n = 19. (B) γ-ENaC 50kD species vs urine protein/creatinine ratio, p = 0.001, n = 19. (C) γ-ENaC 50kD species vs urine protein/creatinine ratio, p = 0.002, n = 19.

Blood pressure did not correlate with any of the Western blot data. Proteinuria, expressed as urinary protein/creatinine ratio, was positively correlated with NKCC2 S130, γ-ENaC 50kD and γ-ENaC 75kD species (Fig 4A–4C). Multiple positive correlations were observed between the intensity of the bands observed in Western blot (Table 4). The more surprising included a positive relationship between NKCC2 S130 phosphorylation and the intensity of α-ENaC expression (Table 4). The association between total NKCC2 and α-ENaC was weaker.

**Table 4:** Linear regression analysis of sodium transporter densitometry as a ratio with CD9.

	NKCC2 pS130	NKCC2 pT101/105	NKCC2	NCC T60	NCC	α-ENaC	γ-ENaC 75kD	γ-ENaC 50kD
pS130			0.428^a^ 0.001			0.621 <0.0001		
pT101/105			0.246 0.019	0.422 0.001	0.320 0.006			
NKCC2	0.428 0.001	0.246 0.019				0.498 0.0002	0.248 0.018	
pT60		0.422 0.001			0.774 <0.0001			
NCC		0.320 0.006		0.774 <0.0001				
α-ENaC	0.621 <0.0001		0.498 0.0002				0.399 0.002	0.305 0.008
γ-ENaC 75 kD			0.248 0.018			0.399 0.002		0.868 <0.0001
γ-ENaC 50 kD						0.305 0.008	0.868 <0.0001	

^a^Linear regression data expressed as R^2^ values (upper line) and ‘p’ value (lower line).

All sodium transporter densitometry values used to derive regressions were expressed as a ratio with the densitometry of CD9. N = 22 for each comparison.

## Discussion

Greater phosphorylation of NKCC2 on S130 in the PE group might be predicted to lead to increased sodium reabsorption, particularly with increased total NKCC2 expression. However, phosphorylation of NKCC2 on the SPAK/OSR1 sites T101/105 was less in the PE group, so it becomes difficult to predict the overall effect on NKCC2 activity. Notwithstanding the complexity, a role for increased co-transporter activity by NKCC2 in the pathogenesis of PE appears consistent with the observation that in women with antenatal pre-eclampsia, postnatal frusemide is associated with a reduced requirement for antihypertensive therapy in hospital [29], although frusemide is not specific for NKCC2 alone.

Although there is no data concerning the mechanism whereby pS130 increases *in vivo*, as outlined earlier, phosphorylation of NKCC2 on S130 can be mediated by AMPK [27] or PKA [10] *in vitro*. Whether phosphorylation of S130 can be used to infer activation of AMPK or PKA in the sections of the renal tubule expressing NKCC2 is not established. Phosphorylation of specific phosphosites in target proteins is often used to assess activity of a protein kinase in tissues and cells, one of the best examples being AMPK where phosphorylation of S79 in acetyl CoA carboxylase is widely used as an indicator of AMPK activity [30]. This is an intriguing possibility for future study.

The data for NCC were unexpected, with less phosphorylation on the putative SPAK/OSR1 site Thr60 in the PE group compared with the others. There was a trend to reduced chloride in the urine of women with PE, which could affect WNK-SPAK-OSR1 signalling [31] but the data is not reliable due to the relatively high end cut-off for the assays of urinary chloride excretion. Moreover, intracellular chloride is generally considered an inhibitor of the WNK-SPAK-OSR1 pathway in the distal convoluted tubule [31], so reduced chloride in the distal convoluted tubule would be expected to increase NCC phosphorylation. The change might be due to unknown alterations in humoral regulation, reducing WNK-SPAK-OSR1 signalling in the thick ascending limb and the distal convoluted tubule.

Both α and γ-ENaC species were increased in this study. The greater expression of the α subunit was marked, but requires careful interpretation. The antibody used was directed at the N-terminus, so that the commonly cleaved species would probably have been missed as they were below the molecular weight where quantitation was performed. In effect, the data shows that women with pre-eclampsia have more uncleaved α-ENaC than controls. Neilsen et al, by contrast, found that most pregnant women had a 70–75 kD species as well as a 50 kD species using an antibody able to detect cleaved species [19]. In their study, the 50 kD species was significantly increased in both NP and PE groups compared with NC. As they state, the 50 kD species likely represents a double furin-cleaved species. This would not have been detected in the current study. The 50 kD species noted in the current study is novel and not predicted by current evidence. Another interesting finding was the correlation between α-ENaC and phosphorylation of NKCC2 on S130. At present, there is no mechanism known to link these events.

Cleaved forms of γ-ENaC were significantly more common in urinary uEVs from women with PE than in normal pregnancy. This is consistent with a previous study showing that patients with pre-eclampsia have more urinary plasmin, and that amiloride-sensitive inward currents in M1 cells increased with exposure to urine from pre-eclampsia patients [32]. Interestingly, the presence of the 75kD and 50kD species of γ-ENaC correlated with the degree of proteinuria. A similar phenomenon has been noted with other forms of proteinuria, where proteases present in the urine are considered to contribute to fragmentation of the γ subunit [14, 15].

There are several areas where this study differs from previous work. Firstly, use of CD9 as a loading control for urinary uEVs, rather that urine creatinine as others have reported [19], requires justification. Salih et al have recently reported that secretion of CD9 is consistent with urinary creatinine excretion [33]. Given the high purity of uEVs obtained from urine by Pisitkun et al [20] use of CD9 appears to be an easier, more direct, and likely more consistent marker for exosome loading that urine creatinine.

A second issue with study of urinary uEVs is how well they reflect expression and phosphorylation of proteins in the kidney. In studies of Gitleman’s and Bartter’s syndromes, there is evidence that urinary uEVs reflect the differences seen in the kidney compared with normal [34]. As reviewed by Salih et al [35], the ability of urinary uEVs to reflect more subtle changes has been addressed in animal and human studies. Esteva-Font et al showed that expression of NKCC2 and NCC correlated well with their expression in the kidney in two rat models of altered salt handling [36]. Qi et al showed that a 20-fold increase in a peptide from γ-ENaC could be identified in urinary uEVs from individuals with increased aldosterone, using mass spectrometry [37]. Wolley et al were able to demonstrate changes in expression and phosphorylation of NCC following mineralocorticoid administration [23]. Finally, Zachar et al reported that expression of the γ subunit of ENaC was present in urinary uEVs, but that the urinary uEVs contained lower molecular weight “cleaved” forms compared with blots of whole kidney cortex [38]. The data suggest that expression of NKCC2 and NCC reflects their abundance in the kidney, and that altered phosphorylation of NCC can be demonstrated in uEVs. There is little data concerning α-ENaC, but expression of γ-ENaC in uEVs seems biased towards smaller forms, possibly reflecting recent expression at the cell surface compared with γ-ENaC seen in blots of kidney cortex, which will contain molecules not at the cell surface.

## Conclusions

This is the first study to identify changes in expression of ENaC and phosphorylation of NCC and NKCC2 in PE. NKCC2 phosphorylation on Ser130 increases its co-transporter activity. Phosphorylation of NCC was less in the PE group, and this is predicted to reduce its activity. The effect might be due to reduced sodium flowing into the distal convoluted tubule due to the increased upstream activity of NKCC2 in the PE group. The changes in expression of the α and γ subunits of ENaC are novel, with the presence of truncated forms of γ-ENaC and greater expression of α-ENaC predicted to increase sodium uptake. This study, therefore, identifies NKCC2 as a major contributor to sodium retention in pre-eclampsia, with ENaC likely to have a lesser role because of its smaller capacity.

## Supporting information

S1 Fig Reproducibility of Western blot data in a normal control subject. Assays were performed on different days using a single preparation from a single urine sample. The uEVs were thawed on three separate occasions. (A) Blots from three assays. (B) Densitometric results expressed as mean + 1 S.D. n = 3.
(TIFF)

## References

[1] Say L, Chou D, Gemmill A, Tuncalp O, Moller AB, Daniels J, et al Global causes of maternal death: a WHO systematic analysis. Lancet Glob Health. 2014;2(6):e323–33. 10.1016/S2214-109X(14)70227-X . 25103301

[2] Tranquilli AL, Dekker G, Magee L, Roberts J, Sibai BM, Steyn W, et al The classification, diagnosis and management of the hypertensive disorders of pregnancy: A revised statement from the ISSHP. Pregnancy Hypertens. 2014;4(2):97–104. 10.1016/j.preghy.2014.02.001 . 26104417

[3] Maynard SE, Min JY, Merchan J, Lim KH, Li J, Mondal S, et al Excess placental soluble fms-like tyrosine kinase 1 (sFlt1) may contribute to endothelial dysfunction, hypertension, and proteinuria in preeclampsia. J Clin Invest. 2003;111(5):649–58. 10.1172/JCI17189 ; PubMed Central PMCID: PMCPMC151901. 12618519 PMC151901

[4] Brown MA, Gallery ED, Ross MR, Esber RP. Sodium excretion in normal and hypertensive pregnancy: a prospective study. Am J Obstet Gynecol. 1988;159(2):297–307. . 3044110 10.1016/s0002-9378(88)80071-1

[5] Brown MA, Wang J, Whitworth JA. The renin-angiotensin-aldosterone system in pre-eclampsia. Clin Exp Hypertens. 1997;19(5–6):713–26. 10.3109/10641969709083181 . 9247750

[6] Lifton RP, Gharavi AG, Geller DS. Molecular mechanisms of human hypertension. Cell. 2001;104(4):545–56. . 11239411 10.1016/s0092-8674(01)00241-0

[7] Richardson C, Alessi DR. The regulation of salt transport and blood pressure by the WNK-SPAK/OSR1 signalling pathway. J Cell Sci. 2008;121(Pt 20):3293–304. 10.1242/jcs.029223 . 18843116

[8] Gimenez I, Forbush B. Regulatory phosphorylation sites in the NH2 terminus of the renal Na-K-Cl cotransporter (NKCC2). Am J Physiol Renal Physiol. 2005;289(6):F1341–5. 10.1152/ajprenal.00214.2005 . 16077079

[9] Richardson C, Sakamoto K, de los Heros P, Deak M, Campbell DG, Prescott AR, et al Regulation of the NKCC2 ion cotransporter by SPAK-OSR1-dependent and -independent pathways. J Cell Sci. 2011;124(Pt 5):789–800. 10.1242/jcs.077230 ; PubMed Central PMCID: PMCPMC3114804. 21321328 PMC3114804

[10] Gunaratne R, Braucht DW, Rinschen MM, Chou CL, Hoffert JD, Pisitkun T, et al Quantitative phosphoproteomic analysis reveals cAMP/vasopressin-dependent signaling pathways in native renal thick ascending limb cells. Proc Natl Acad Sci U S A. 2010;107(35):15653–8. 10.1073/pnas.1007424107 ; PubMed Central PMCID: PMCPMC2932563. 20713729 PMC2932563

[11] Fraser SA, Gimenez I, Cook N, Jennings I, Katerelos M, Katsis F, et al Regulation of the renal-specific Na+-K+-2Cl- co-transporter NKCC2 by AMP-activated protein kinase (AMPK). Biochem J. 2007;405(1):85–93. 10.1042/BJ20061850 ; PubMed Central PMCID: PMCPMC1925243. 17341212 PMC1925243

[12] Richardson C, Rafiqi FH, Karlsson HK, Moleleki N, Vandewalle A, Campbell DG, et al Activation of the thiazide-sensitive Na+-Cl- cotransporter by the WNK-regulated kinases SPAK and OSR1. J Cell Sci. 2008;121(Pt 5):675–84. 10.1242/jcs.025312 . 18270262

[13] Vallet V, Chraibi A, Gaeggeler HP, Horisberger JD, Rossier BC. An epithelial serine protease activates the amiloride-sensitive sodium channel. Nature. 1997;389(6651):607–10. 10.1038/39329 . 9335501

[14] Passero CJ, Mueller GM, Rondon-Berrios H, Tofovic SP, Hughey RP, Kleyman TR. Plasmin activates epithelial Na+ channels by cleaving the gamma subunit. J Biol Chem. 2008;283(52):36586–91. 10.1074/jbc.M805676200 ; PubMed Central PMCID: PMCPMC2605981. 18981180 PMC2605981

[15] Svenningsen P, Friis UG, Bistrup C, Buhl KB, Jensen BL, Skott O. Physiological regulation of epithelial sodium channel by proteolysis. Curr Opin Nephrol Hypertens. 2011;20(5):529–33. 10.1097/MNH.0b013e328348bcc7 . 21670672

[16] Kleyman TR, Carattino MD, Hughey RP. ENaC at the cutting edge: regulation of epithelial sodium channels by proteases. J Biol Chem. 2009;284(31):20447–51. Epub 2009/04/30. 10.1074/jbc.R800083200 ; PubMed Central PMCID: PMCPMC2742807. 19401469 PMC2742807

[17] Davies MR, Gleich K, Katerelos M, Lee M, Mount PF, Power DA. The Thiazide-Sensitive Co-Transporter Promotes the Development of Sodium Retention in Mice with Diet-Induced Obesity. Kidney Blood Press Res. 2015;40(5):509–19. Epub 2015/09/30. 10.1159/000368527 . 26418861

[18] Davies M, Fraser SA, Galic S, Choy SW, Katerelos M, Gleich K, et al Novel mechanisms of Na+ retention in obesity: phosphorylation of NKCC2 and regulation of SPAK/OSR1 by AMPK. Am J Physiol Renal Physiol. 2014;307(1):F96–F106. Epub 2014/05/09. 10.1152/ajprenal.00524.2013 . 24808538

[19] Nielsen MR, Frederiksen-Moller B, Zachar R, Jorgensen JS, Hansen MR, Ydegaard R, et al Urine exosomes from healthy and hypertensive pregnancies display elevated level of alpha-subunit and cleaved alpha- and gamma-subunits of the epithelial sodium channel-ENaC. Pflugers Arch. 2017 10.1007/s00424-017-1977-z . 28405801

[20] Pisitkun T, Shen RF, Knepper MA. Identification and proteomic profiling of exosomes in human urine. Proc Natl Acad Sci U S A. 2004;101(36):13368–73. 10.1073/pnas.0403453101 ; PubMed Central PMCID: PMCPMC516573. 15326289 PMC516573

[21] van der Lubbe N, Jansen PM, Salih M, Fenton RA, van den Meiracker AH, Danser AH, et al The phosphorylated sodium chloride cotransporter in urinary exosomes is superior to prostasin as a marker for aldosteronism. Hypertension. 2012;60(3):741–8. 10.1161/HYPERTENSIONAHA.112.198135 . 22851731

[22] Andersen H, Friis UG, Hansen PB, Svenningsen P, Henriksen JE, Jensen BL. Diabetic nephropathy is associated with increased urine excretion of proteases plasmin, prostasin and urokinase and activation of amiloride-sensitive current in collecting duct cells. Nephrol Dial Transplant. 2015;30(5):781–9. 10.1093/ndt/gfu402 . 25609736

[23] Wolley MJ, Wu A, Xu S, Gordon RD, Fenton RA, Stowasser M. In Primary Aldosteronism, Mineralocorticoids Influence Exosomal Sodium-Chloride Cotransporter Abundance. J Am Soc Nephrol. 2017;28(1):56–63. 10.1681/ASN.2015111221 . 27381844 PMC5198275

[24] Lowe SA, Bowyer L, Lust K, McMahon LP, Morton MR, North RA, et al The SOMANZ Guidelines for the Management of Hypertensive Disorders of Pregnancy 2014. Aust N Z J Obstet Gynaecol. 2015;55(1):11–6. 10.1111/ajo.12253 . 25308532

[25] Thery C, Amigorena S, Raposo G, Clayton A. Isolation and characterization of exosomes from cell culture supernatants and biological fluids. Curr Protoc Cell Biol. 2006;Chapter 3:Unit 3 22. 10.1002/0471143030.cb0322s30 . 18228490

[26] Fraser SA, Choy SW, Pastor-Soler NM, Li H, Davies MR, Cook N, et al AMPK couples plasma renin to cellular metabolism by phosphorylation of ACC1. Am J Physiol Renal Physiol. 2013;305(5):F679–90. 10.1152/ajprenal.00407.2012 ; PubMed Central PMCID: PMCPMC3761205. 23785098 PMC3761205

[27] Cook N, Fraser SA, Katerelos M, Katsis F, Gleich K, Mount PF, et al Low salt concentrations activate AMP-activated protein kinase in mouse macula densa cells. Am J Physiol Renal Physiol. 2009;296(4):F801–9. 10.1152/ajprenal.90372.2008 . 19176702

[28] Mathivanan S, Simpson RJ. ExoCarta: A compendium of exosomal proteins and RNA. Proteomics. 2009;9(21):4997–5000. 10.1002/pmic.200900351 . 19810033

[29] Magee L, von Dadelszen P. Prevention and treatment of postpartum hypertension. Cochrane Database Syst Rev. 2013;(4):CD004351 10.1002/14651858.CD004351.pub3 . 23633317 PMC11999663

[30] Steinberg GR, Kemp BE. AMPK in Health and Disease. Physiol Rev. 2009;89(3):1025–78. Epub 2009/07/09. 10.1152/physrev.00011.2008 . 19584320

[31] Terker AS, Zhang C, Erspamer KJ, Gamba G, Yang CL, Ellison DH. Unique chloride-sensing properties of WNK4 permit the distal nephron to modulate potassium homeostasis. Kidney Int. 2016;89(1):127–34. Epub 2015/10/01. 10.1038/ki.2015.289 ; PubMed Central PMCID: PMCPMC4814375. 26422504 PMC4814375

[32] Buhl KB, Friis UG, Svenningsen P, Gulaveerasingam A, Ovesen P, Frederiksen-Moller B, et al Urinary plasmin activates collecting duct ENaC current in preeclampsia. Hypertension. 2012;60(5):1346–51. 10.1161/HYPERTENSIONAHA.112.198879 . 22987920

[33] Salih M, Fenton RA, Knipscheer J, Janssen JW, Vredenbregt-van den Berg MS, Jenster G, et al An immunoassay for urinary extracellular vesicles. Am J Physiol Renal Physiol. 2016;310(8):F796–F801. 10.1152/ajprenal.00463.2015 . 26823283

[34] Corbetta S, Raimondo F, Tedeschi S, Syren ML, Rebora P, Savoia A, et al Urinary exosomes in the diagnosis of Gitelman and Bartter syndromes. Nephrol Dial Transplant. 2015;30(4):621–30. 10.1093/ndt/gfu362 . 25422309

[35] Salih M, Fenton RA, Zietse R, Hoorn EJ. Urinary extracellular vesicles as markers to assess kidney sodium transport. Curr Opin Nephrol Hypertens. 2016;25(2):67–72. 10.1097/MNH.0000000000000192 . 26717312

[36] Esteva-Font C, Wang X, Ars E, Guillen-Gomez E, Sans L, Gonzalez Saavedra I, et al Are sodium transporters in urinary exosomes reliable markers of tubular sodium reabsorption in hypertensive patients? Nephron Physiol. 2010;114(3):p25–34. 10.1159/000274468 ; PubMed Central PMCID: PMCPMC2840242. 20068364 PMC2840242

[37] Qi Y, Wang X, Rose KL, MacDonald WH, Zhang B, Schey KL, et al Activation of the Endogenous Renin-Angiotensin-Aldosterone System or Aldosterone Administration Increases Urinary Exosomal Sodium Channel Excretion. J Am Soc Nephrol. 2016;27(2):646–56. 10.1681/ASN.2014111137 ; PubMed Central PMCID: PMCPMC4731116. 26113616 PMC4731116

[38] Zachar RM, Skjodt K, Marcussen N, Walter S, Toft A, Nielsen MR, et al The epithelial sodium channel gamma-subunit is processed proteolytically in human kidney. J Am Soc Nephrol. 2015;26(1):95–106. 10.1681/ASN.2013111173 ; PubMed Central PMCID: PMCPMC4279735. 25060057 PMC4279735

