# Supplementary figures and images for: Pre-eclampsia is associated with altered expression of the renal sodium transporters NKCC2, NCC and ENaC in urinary extracellular vesicles
Source: PLoS One. 2018 Sep 24;13(9):e0204514. doi: 10.1371/journal.pone.0204514 (PMC6152984; doi:10.1371/journal.pone.0204514)

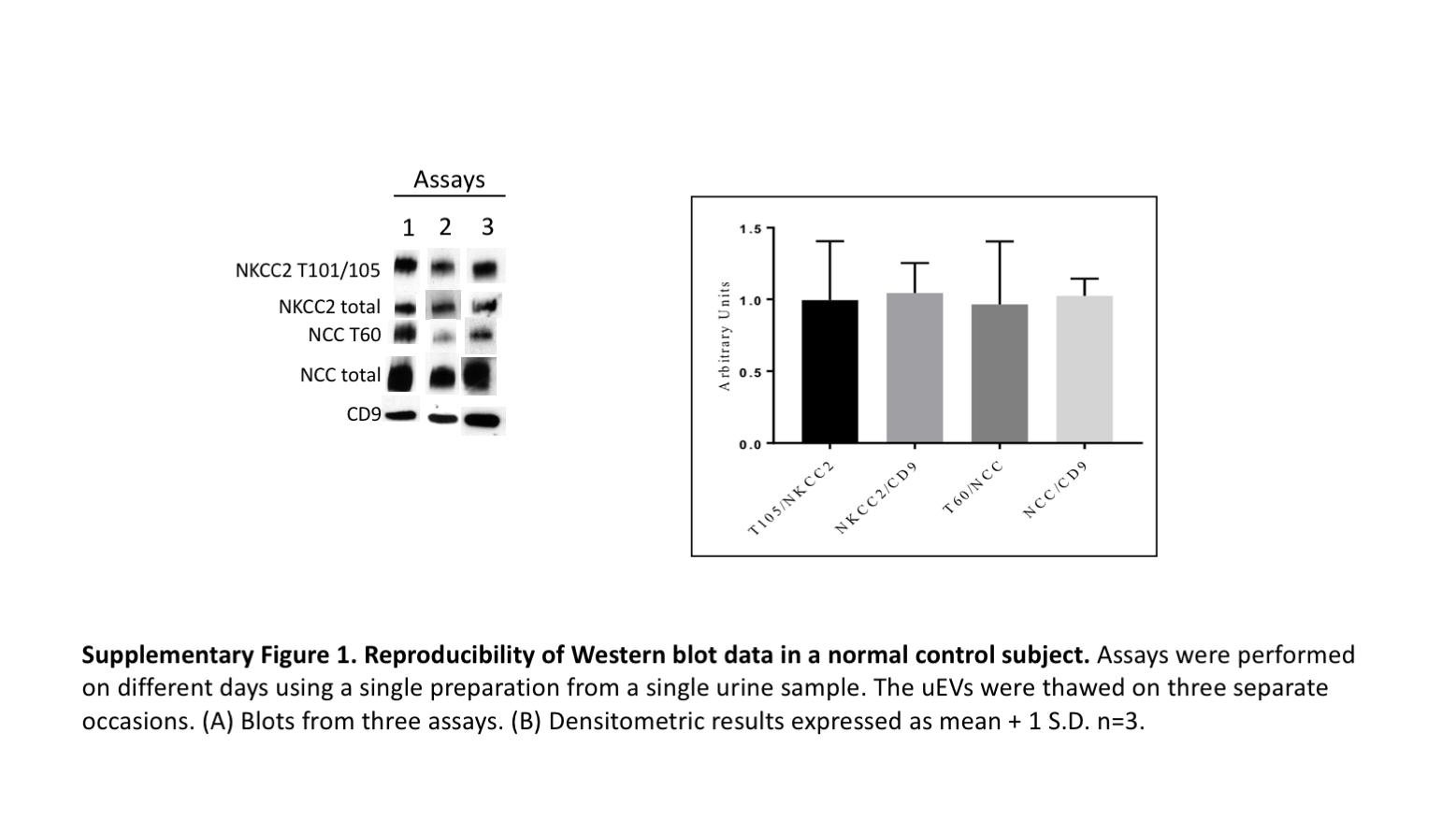

Supplement: S1 Fig — Assays were performed on different days using a single preparation from a single urine sample. The uEVs were thawed on three separate occasions. (A) Blots from three assays. (B) Densitometric results expressed as mean + 1 S.D. n = 3. (TIFF) [file pone.0204514.s001.tiff]
